# Is Handgrip Strength a Valid and Reliable Measure in Older Adults?

**DOI:** 10.1093/geroni/igaa057.609

**Published:** 2020-12-16

**Authors:** Jefferson Spicher, Amy Silva-Smith, Melissa Benton

**Affiliations:** University of Colorado Colorado Springs, Colorado Springs, Colorado, United States

## Abstract

Handgrip strength is related to mortality, disability, functional independence, and quality of life in older adults, and cut points for diagnosis of sarcopenia have been proposed. However, there is no standardized procedure or device, so measurement may not be accurate. To assess validity and reliability we compared hydraulic (HD) versus digital (DD) handgrip dynamometers. Sixty-seven older (76.2 ± 0.9 years) men (n=34) and women (n=33) completed two measurements on sequential days (T1, T2) using both devices in random order. Participants sat in a chair with the device held in their dominant hand, their arm supported on a table or other stable surface, their wrist in a neutral position, and their elbow bent at a 90° angle. To avoid muscle fatigue that has been attributed to multiple attempts, participants squeezed the device one time as hard as possible for 3 seconds. Strong (p<0.001) intraclass correlations were observed for both devices (HD=0.98; SS=0.96) indicating good reliability. However, there were significant differences between devices and between measurements. Strength measured with HD was greater than DD at T1 (27.4 ± 1.4 vs. 23.4 ± 1.1 kg, p<0.001) and T2 (25.3 ± 1.4 vs. 21.8 ± 1.2 kg, p<0.001). Day-to-day measurements were also significantly different. Between T1 and T2 strength decreased 8% with HD (p<0.001) and decreased 7% with DD (p=0.001). In this group of older adults, significant differences in handgrip strength were observed between devices and timepoints indicating poor validity. As a diagnostic tool, standardization is needed for handgrip measurement procedures to improve accuracy.

